# Supplementary material for: Wind conditions influence breeding season movements in a nomadic polygynous shorebird
Source: Proc Biol Sci. 2020 Feb 12;287(1920):20192789. doi: 10.1098/rspb.2019.2789 (PMC7031675; doi:10.1098/rspb.2019.2789)
Supplement: Supplementary Tables and Figures [file rspb20192789supp1.pdf]

Wind conditions influence breeding season movements in a nomadic polygynous shorebird

Johannes Krietsch, Mihai Valcu and Bart Kempenaers

Electronic supplementary material

Contents

Supplementary Tables ... 2
Table S1. ... 2
Table S2. ... 3
Table S3. ... 4
Table S4. ... 5
Table S5. ... 6
Table S6. ... 7
Table S7. ... 8
Table S8. ... 9
Table S9. ... 10
Table S10. ... 11
Supplementary Figures ... 12
Figure S1. ... 12
Figure S2. ... 13
Figure S3. ... 14
Figure S4. ... 15
Figure S5. ... 16
Figure S6. ... 17

Supplementary Tables

Table S1. Most likely flight altitude based on a linear mixed-effect model of ground speed (m/s) explained by wind support and crosswind for each altitude with track ID as random effect. The altitude with maximal wind support (maxWs) was calculated based on maximum wind support (see Methods). To control for temporal autocorrelation we used a moving-average correlation structure. Models were fitted with maximum likelihood and ranked based on the AIC criterion. For the summary statistics of the best fitting model see Table S2

altitude	AICc	delta AIC
750 m	14997.03	0
1500 m	15002.04	5.0
2250 m	15023.86	26.8
maxWs	15026.14	29.1
100 m	15039.41	42.4
3000 m	15046.22	49.2
10 m	15047.66	50.6

Table S2. Influence of wind support and crosswind on the ground and air speed of male pectoral sandpipers (n = 85 tracks). Shown are results from a linear mixed-effect model with ground speed (m/s) or air speed (m/s) as the dependent variable, with wind support and crosswind (at ~750 m altitude) as explanatory variables and with track ID as random intercept. To control for temporal autocorrelation we used a moving-average correlation structure.

dependent variable	term	estimate	s.e.	statistic	*p*
ground speed	intercept	14.63	0.28	53.10	
	wind support	0.31	0.03	9.17	<0.001
	crosswind	-0.06	0.03	-1.72	0.27
	wind support x crosswind	-0.02	0.00	-5.61	<0.001
	track ID (variance)	0.00			
	residual variance	5.18			
air speed	Intercept	14.73	0.24	61.06	
	wind support	-0.58	0.02	-24.32	<0.001
	crosswind	0.26	0.03	8.64	<0.001
	track ID (variance)	1.13			
	residual variance	4.07			

Table S3. The relationship between track length and mean wind support (WS, in m/s) during the first half of the flight (n = 85 tracks). We fitted a linear model using wind data at ~750 m altitude and included category as fixed factors to correct for the effect of different potential flight lengths based on direction.

**term**	**estimate**	**s.e.**	**statistic**	***p***
intercept	1150.71	57.95	19.86	
category (loop)	299.47	163.50	1.83	0.22
category (west)	315.35	115.53	2.73	0.028
mean WS first half	21.73	9.85	2.21	0.030

Table S4. Influence of wind direction (degrees) in combination with wind speed (m/s) during the first 50 km on the initial flight direction (mean over the first 50 km) for male pectoral sandpipers that left Utqiaġvik, Alaska (n = 85 tracks). Shown are results from a linear mixed-effect model with initial direction (degrees) as the dependent variable, with an interaction of wind direction and wind speed (at ~750 m altitude) as explanatory variables and with date (night in a given year) as random intercept.

**model**	**term**	**estimate**	**s.e.**	**statistic**	***p***
continuous wind speed	intercept	18.35	10.06	1.82	
	wind direction	-0.01	0.09	-0.13	1.00
	wind speed	2.31	1.63	1.41	0.39
	wind direction * wind speed	0.07	0.02	4.16	<0.001
	day (variance)	11.26			
	residual variance	40.86			
categorised wind speed	Intercept	27.18	9.86	2.76	
	wind direction	0.65	0.11	5.78	<0.001
	wind speed low	4.86	12.92	0.38	0.98
	wind direction * wind speed low	-0.55	0.14	-4.05	<0.001
	day (variance)	21.15			
	residual variance	38.07			

Table S5. Relationship between the final flight direction (the direction from the departure to the arrival location) and the initial flight direction (average of the first 50 km). Shown are results from a linear mixed-effect model with final flight direction (degrees) as the dependent variable, initial flight direction as explanatory variable and with date (night in a given year) as random intercept.

term	estimate	s.e.	statistic	*p*
intercept	26.30	10.70	2.46	
initial direction	1.07	0.13	8.51	<0.001
day (variance)	42.17			
residual variance	48.31			

Table S6. Results of a binominal model of the number of departures of male pectoral sandpipers going west (n = 36) and east (n = 45) against initial delta wind support (mean wind support for going west minus mean wind support for going east for the first 50 km). Date (the night of departure in each year) is included as random effect. The wind support (at ~750 m altitude) for every night was estimated by using all tracks of birds that went either west or east, shifting their departure to the average departure hour (22:30 h) for each night (see Methods).

term	estimate	s.e.	statistic	*p*
intercept	-1.37	0.61	-2.23	
initial delta wind support	0.12	0.04	2.80	0.009
day (variance)	0.67			

Table S7. Results of a linear mixed-effect model of relative wind speed explained by relative track length and wind speed (m/s) using track ID as random effect (n = 85 tracks). Relative wind support is defined as wind support (calculated based on wind data at ~750 m altitude, see Methods), divided by wind speed at each location. A relative wind support of one corresponds to a heading that is identical to the direction of the wind, independent of the wind speed; a relative wind support of minus one corresponds to a bird flying entirely against the wind. To allow comparison between tracks of different length (min = 584 km; max = 2609 km), we show relative track length (dividing by total track length).

**term**	**estimate**	**s.e.**	**statistic**	***p***
intercept	0.16	0.08	2.09	
relative track length	-0.38	0.08	-4.44	<0.001
wind speed	0.03	0.00	5.63	<0.001
track ID (variance)	0.35			
residual variance	0.59			

Table S8. Results of a linear model with mean wind support by track as response variable and category (east, west and loop) and year as fixed factors. Wind support between each flight category was compared with a post-hoc Tukey test.

**term**	**estimate**	**s.e.**	**statistic**	***p***
category (west - loop)	4.23	1.40	3.02	0.009
category (west - east)	6.58	0.81	8.17	<0.001
category (loop - east)	2.35	1.37	1.72	0.20
year (2014)	0.87	0.77	1.12	0.58

Table S9. Differences in wind support depending on shifts in departure date and time. Results from linear mixed-effect models of delta wind support (real-time track minus time-shifted track) of male pectoral sandpipers separated in birds going west (n = 33), east (n = 45) or loops (n = 7). Each track was shifted in 24 h intervals for ±5 days and in 1 h intervals for ±12 h. *p*-values indicate whether the difference in wind support between the observed and shifted time was significantly different from zero, i.e. whether birds would on average have gained or lost wind support by departing at a different day or time. We used track ID and day (night of departure in each year) as random effects.

**model**	**term**	**estimate**	**s.e.**	**statistic**	***p***
days	shift to later	-0.01	0.01	-1.19	0.65
	category (east)	0.59	1.31	0.45	0.99
	category (loop)	0.47	2.47	0.19	1.00
	category (west)	3.55	1.54	2.31	0.09
	shift to earlier	1.05	0.91	1.15	0.69
	night (variance)	5.04			
	track ID (variance)	4.08			
	residual variance	5.67			
hours	shift to later	0.00	0.01	-0.47	0.99
	category (east)	-0.02	0.18	-0.09	1.00
	category (loop)	-0.04	0.31	-0.12	1.00
	category (west)	0.16	0.20	0.78	0.90
	shift to earlier	0.06	0.12	0.54	0.98
	night (variance)	0.59			
	track ID (variance)	0.62			
	residual variance	1.22			

Table S10. Results from linear mixed-effect models of (a) the difference in wind support (delta WS, in m/s, at ~750 m altitude) between the actual track and the shortest route and (b) the difference in flight time (delta time) for the actual versus the shortest route in relation to track category. Loop flights are defined as tracks with high straightness (median = 7.8). Westward and eastward flights are defined as tracks directed toward their respective destination with low straightness (see Methods). In both models, date (the night of departure in each year) was included as random effect.

**dependent variable**	**term**	**estimate**	**s.e.**	**statistic**	***p***
delta WS	category (west)	-1.53	0.87	-1.75	0.20
	category (loop)	3.51	1.31	2.69	0.020
	category (east)	2.05	0.76	2.71	0.019
	day (variance)	3.01			
	residual variance	2.58			
delta time	category (west)	0.53	1.22	0.43	0.96
	category (loop)	23.28	2.43	9.58	<0.001
	category (east)	2.57	1.02	2.53	0.034
	day (variance)	1.73			
	residual variance	6.11			

Supplementary Figures

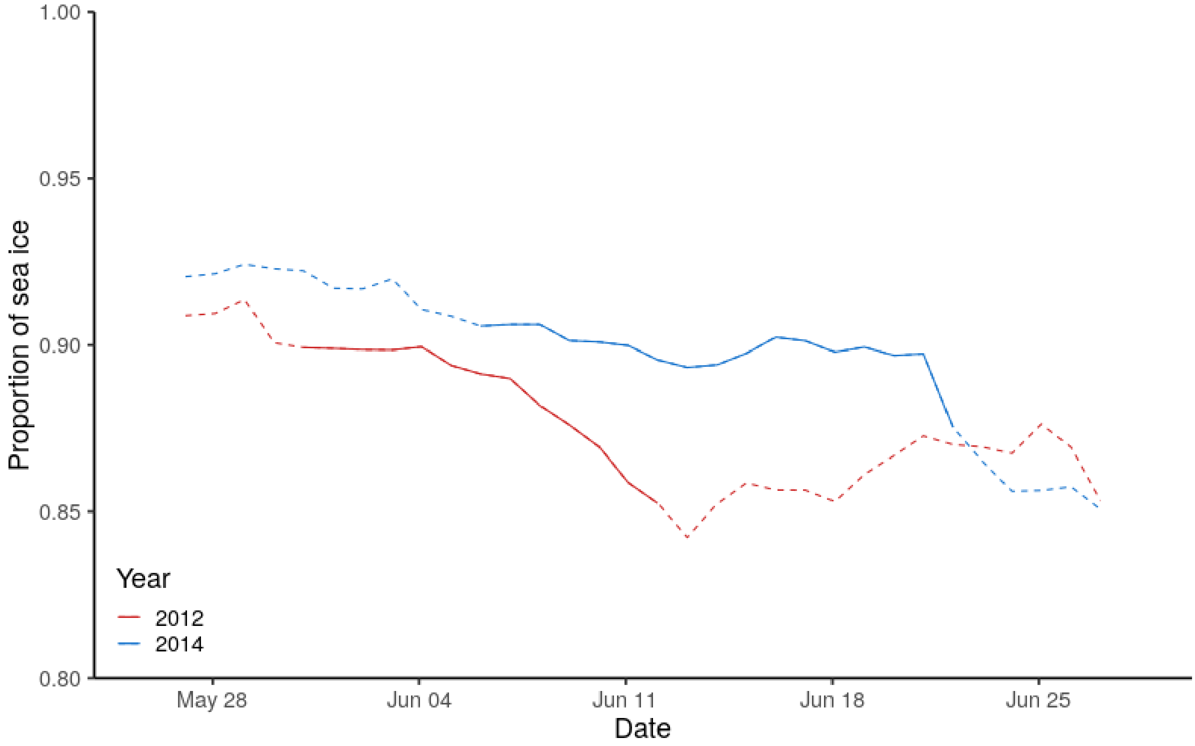

Figure S1. Proportion of sea-ice cover within the convex hull encompassing all tracks of male pectoral sandpipers (n = 85) over the ocean (see Methods) between 27 May and 27 June in 2012 (red) and 2014 (blue). Solid lines indicate the period between the first and last departure in each year. Sea ice data were obtained from the National Snow & Ice Data Centre [53], which provides daily information on sea ice cover with a 4 km resolution. Most of the ice-free ocean was located west or south-west of Banks Island.

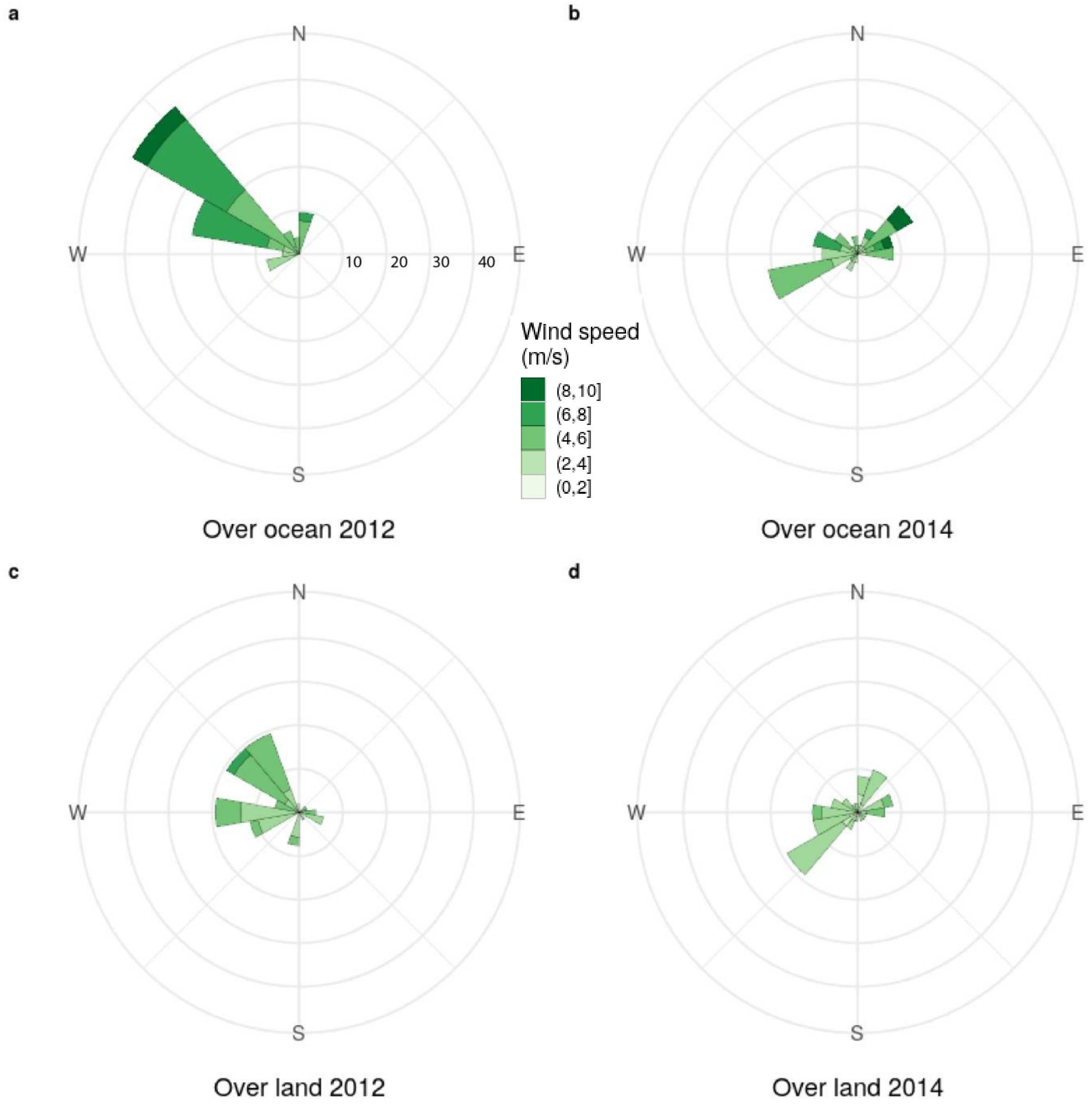

Figure S2. Prevailing wind direction (in % of the time) and wind speed at ~750 m (most likely flight altitude) within the area of all male tracks (n = 85) over the ocean (a, b) and over land (c, d) on days with actual departures in 2012 (a, c) and 2014 (b, d). For every day the mean wind speed and direction within the area was calculated (see Methods). Over the ocean, wind direction did not vary significantly in mean between years (Rao's Tests for Equality of Polar Vectors = 0.02, $p = 0.87$), but in dispersion (Rao's Test for Equality of Dispersions = 106.94, $p < 0.001$). In general, wind speed was higher over the ocean than over land (difference: 1.61 m/s ± 0.37 s.e., $p < 0.001$) and higher in 2012 compared to 2014 (difference: 1.15 m/s ± 0.37 s.e., $p = 0.003$; linear mixed-effect models with departure night (scaled by year) as random effect). In the legend, a square bracket indicates that the value is included, a round bracket indicates any value higher than the indicated value.

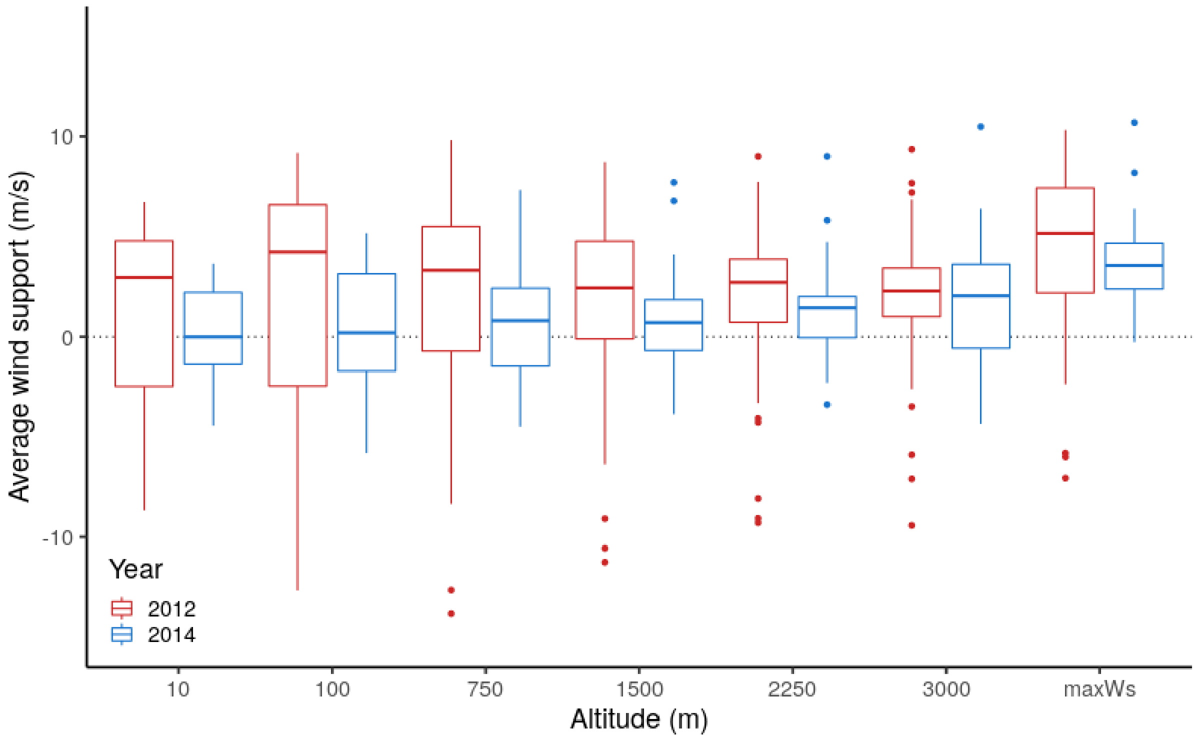

Figure S3. Mean wind support for male pectoral sandpipers that departed around Utqiaġvik, Alaska in 2012 (n = 49) and 2014 (n = 36) for different flight altitudes. The altitude with maximal wind support (maxWs) was calculated based on the highest mean wind support in each 1 h flight bout (from among the six fixed altitudes). Mean wind support was significantly different between 10 m and 2250 m, between 10 m and 3000 m, and between 100 m and 3000 m (based on a linear mixed-effect model with mean wind support as the dependent variable, altitude and year as fixed factors, and individual ID as random intercept; Tukey test to compare different altitudes). The mean wind support of the maxWs altitude was significantly higher than the mean wind support of any fixed altitude (based on the same model).

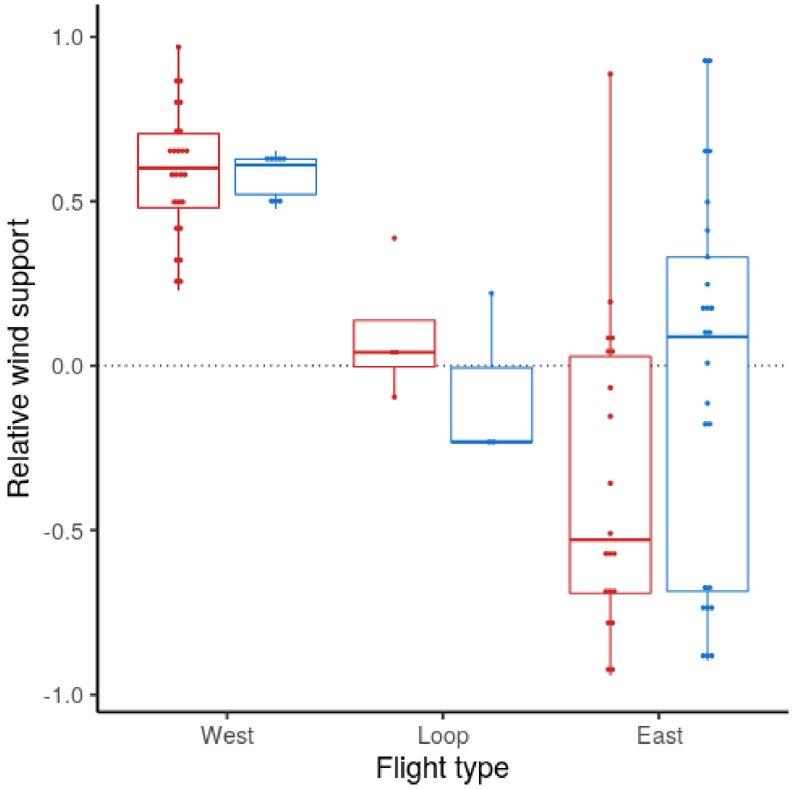

Figure S4. Average relative wind support for the entire flight in relation to flight type and year. Relative wind support is defined as wind support (calculated based on wind data at ~750 m altitude, see Methods), divided by wind speed at each location. Loop flights are defined as tracks with a high straightness (median = 7.8). Westward and eastward flights are defined as tracks directed toward their respective destination with low straightness (see Methods). Shown are box-plots with median (centre line), 25-75th percentile (limits), minimum and maximum values without outliers (whiskers), and outliers (dots). The number above the box indicates the number of tracks in each category.

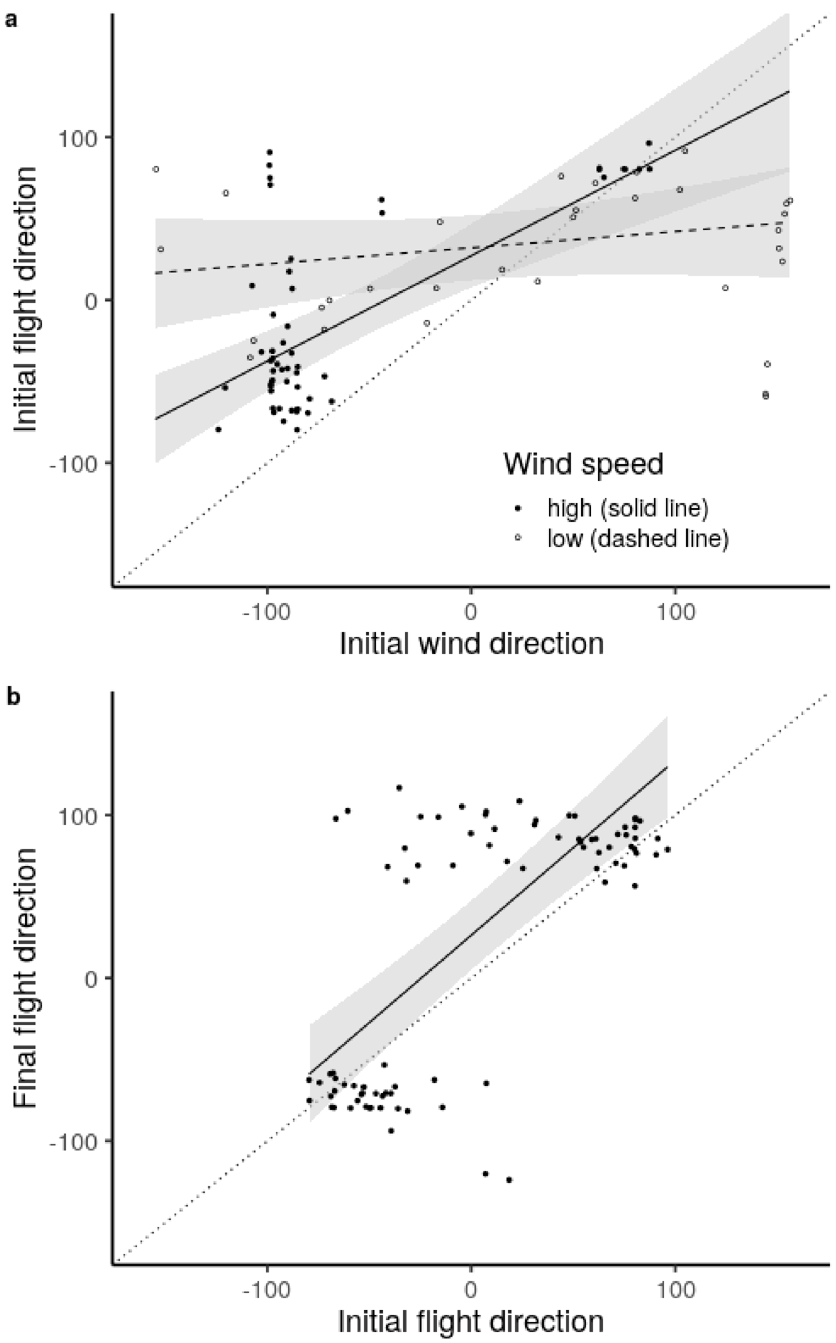

Figure S5. (a) Initial flight direction of male pectoral sandpipers (0 = north, mean of the first 50 km) in relation to the initial wind direction (mean wind direction during the first 50 km of the flight at ~750 m altitude), overall (solid line) and for different wind speeds (low: <5 m/s, high: >5 m/s). Data from 2012 (n = 49) and 2014 (n = 36). (b) Final flight direction (the direction from the departure to the arrival location) in relation to the initial flight direction. Shown are model estimates (lines) and 95% confidence intervals (grey areas). See electronic supplementary materials tables S4-S5 for model descriptions.

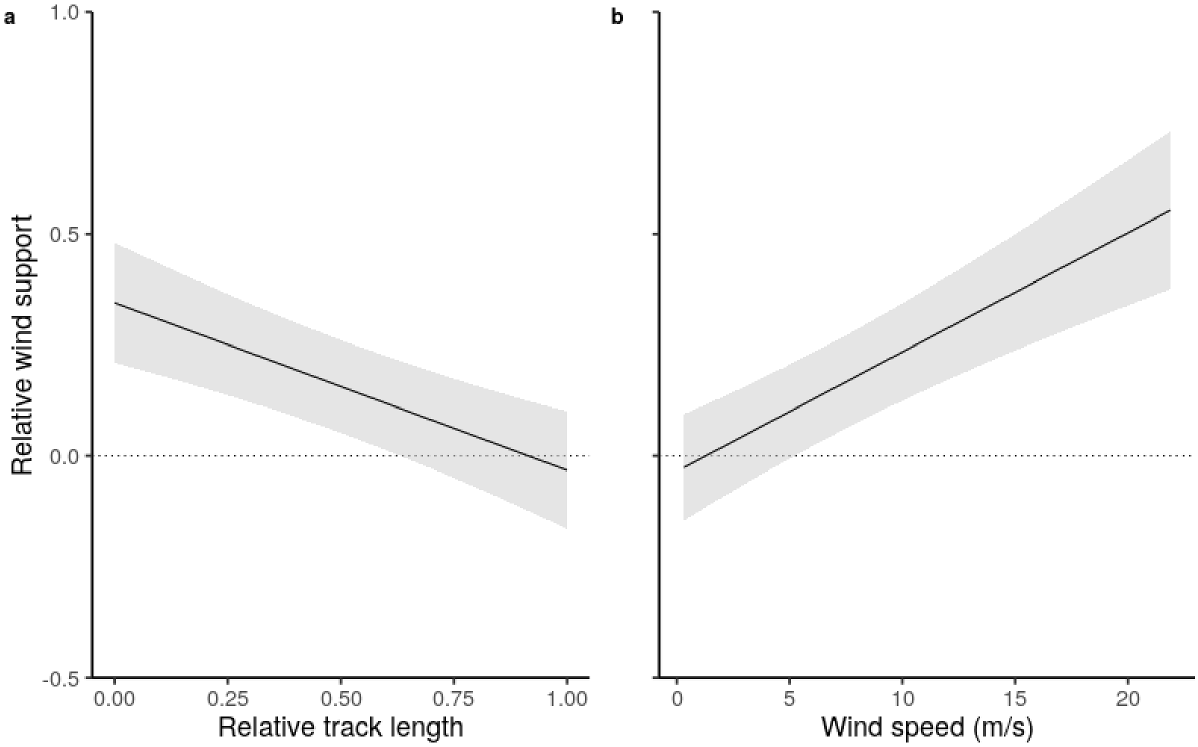

Figure S6. Change in relative wind support for male pectoral sandpipers (**a**) along the track and (**b**) with increasing wind speed. Males departed around Utqiaġvik, Alaska in 2012 (n = 49) and 2014 (n = 36). Relative wind support is defined as wind support (calculated based on wind data at ~750 m altitude; see Methods), divided by wind speed at each location. A relative wind support of one corresponds to a heading identical to the direction of the wind, independent of the wind speed; a relative wind support of minus one corresponds to a bird flying directly against the wind. To allow comparison between tracks of different length, we show relative track length. Shown are model estimates (lines) and 95% confidence intervals (grey areas), see electronic supplementary materials, table S9 for model description.
