# Supplementary material for: Wind conditions influence breeding season movements in a nomadic polygynous shorebird
Source: Proc Biol Sci. 2020 Feb 12;287(1920):20192789. doi: 10.1098/rspb.2019.2789 (PMC7031675; doi:10.1098/rspb.2019.2789)
Supplement: Supplementary tracks [file rspb20192789supp2.html]

 Supplementary tracks 


#### Supplementary Tracks

**Maps of all tracks of individual male pectoral sandpipers that departed the area around Utqiagvik, Alaska in 2012 (n = 49) and 2014 (n = 36)**. Colour indicates wind support (in m/s; green for tailwinds and red for headwinds, see Methods for details). Tracks are shown as minimum convex polygons with 100 km spatial buffer (map scale lower right corner). White arrows at every fifth position indicate the current wind direction and wind speed, in m/s scaled by a factor of 10000), to increase visibility. The grey-blue area indicates suitable breeding habitat within the known breeding range of the pectoral sandpiper [27]. Map projection: polar Lambert azimuthal equal area with longitude origin 156.65� W (Utqiagvik) from Natural Earth (http://www.naturalearthdata.com). Below each map the following information is provided: **ID**, a combination of individual identity and �a� or �b� in case there is more than one track for an individual; **Departure time**, date and time of the first position of the track over the sea; **Flight time**, time from departure time until the last position over sea; **Flight length**, the distance covered between the departure and arrival points; **Detour**, detour in comparison to the shortest distance between the departure and arrival points; **Track straightness**, track length divided by the shortest distance between the departure and arrival location, a straightness of 1 indicates a direct flight between two points; **Mean wind support (WS) and the mean wind support on the shortest route (shortest)**, the mean wind support during the flight at ~750 m altitude and the mean wind support the bird would have experienced had it taken the shortest route (see Methods).

- ID
  :   102081

  Departure time
  :   2012-05-31 00:04:59 AKDT

  Flight time
  :   25.75 hours

  Flight length
  :   1093.14 km

  Detour
  :   100.98 km

  Track straightness
  :   1.1

  WS (shortest)
  :   -4.1 m/s (-2.05 m/s)
- ID
  :   102082

  Departure time
  :   2012-06-03 20:44:41 AKDT

  Flight time
  :   26.5 hours

  Flight length
  :   1878.04 km

  Detour
  :   76.38 km

  Track straightness
  :   1.04

  WS (shortest)
  :   8.88 m/s (7.49 m/s)
- ID
  :   102084

  Departure time
  :   2012-06-03 22:12:54 AKDT

  Flight time
  :   19.5 hours

  Flight length
  :   1067.77 km

  Detour
  :   109.89 km

  Track straightness
  :   1.11

  WS (shortest)
  :   -2.05 m/s (-3.28 m/s)
- ID
  :   102086a

  Departure time
  :   2012-06-02 02:02:19 AKDT

  Flight time
  :   20.75 hours

  Flight length
  :   1215.3 km

  Detour
  :   612.75 km

  Track straightness
  :   2.02

  WS (shortest)
  :   0.3 m/s (-4.35 m/s)
- ID
  :   102086b

  Departure time
  :   2012-06-08 22:02:37 AKDT

  Flight time
  :   24 hours

  Flight length
  :   1813.55 km

  Detour
  :   49.59 km

  Track straightness
  :   1.03

  WS (shortest)
  :   5.33 m/s (6.44 m/s)
- ID
  :   102088

  Departure time
  :   2012-06-08 23:54:25 AKDT

  Flight time
  :   18.75 hours

  Flight length
  :   1402.91 km

  Detour
  :   14.68 km

  Track straightness
  :   1.01

  WS (shortest)
  :   8.88 m/s (10.29 m/s)
- ID
  :   102089

  Departure time
  :   2012-06-04 22:12:44 AKDT

  Flight time
  :   31.75 hours

  Flight length
  :   1827.29 km

  Detour
  :   21.64 km

  Track straightness
  :   1.01

  WS (shortest)
  :   5.82 m/s (5.84 m/s)
- ID
  :   102090

  Departure time
  :   2012-06-04 23:42:25 AKDT

  Flight time
  :   26.89 hours

  Flight length
  :   1686.53 km

  Detour
  :   122.88 km

  Track straightness
  :   1.08

  WS (shortest)
  :   4.81 m/s (4.04 m/s)
- ID
  :   102091

  Departure time
  :   2012-05-31 22:26:12 AKDT

  Flight time
  :   23 hours

  Flight length
  :   1475.87 km

  Detour
  :   20.8 km

  Track straightness
  :   1.01

  WS (shortest)
  :   0.69 m/s (0.61 m/s)
- ID
  :   102092

  Departure time
  :   2012-05-31 22:30:04 AKDT

  Flight time
  :   11.76 hours

  Flight length
  :   819.59 km

  Detour
  :   30.94 km

  Track straightness
  :   1.04

  WS (shortest)
  :   2.94 m/s (3.63 m/s)
- ID
  :   102093

  Departure time
  :   2012-06-08 20:00:44 AKDT

  Flight time
  :   16.2 hours

  Flight length
  :   601.49 km

  Detour
  :   205.12 km

  Track straightness
  :   1.52

  WS (shortest)
  :   -12.66 m/s (-18.45 m/s)
- ID
  :   102094

  Departure time
  :   2012-06-07 00:31:55 AKDT

  Flight time
  :   26.18 hours

  Flight length
  :   1031.9 km

  Detour
  :   480.99 km

  Track straightness
  :   1.87

  WS (shortest)
  :   -6.34 m/s (-10.45 m/s)
- ID
  :   114267

  Departure time
  :   2012-05-30 22:07:52 AKDT

  Flight time
  :   24 hours

  Flight length
  :   1102.36 km

  Detour
  :   10 km

  Track straightness
  :   1.01

  WS (shortest)
  :   -4.64 m/s (-4.27 m/s)
- ID
  :   114270

  Departure time
  :   2012-06-05 01:09:44 AKDT

  Flight time
  :   32.48 hours

  Flight length
  :   1982.99 km

  Detour
  :   137.51 km

  Track straightness
  :   1.07

  WS (shortest)
  :   5.61 m/s (6.25 m/s)
- ID
  :   114272

  Departure time
  :   2012-06-07 16:20:45 AKDT

  Flight time
  :   29.83 hours

  Flight length
  :   1316.36 km

  Detour
  :   231.21 km

  Track straightness
  :   1.21

  WS (shortest)
  :   -7.73 m/s (-12.13 m/s)
- ID
  :   114273a

  Departure time
  :   2012-06-03 18:55:49 AKDT

  Flight time
  :   41.18 hours

  Flight length
  :   2442.07 km

  Detour
  :   1966.63 km

  Track straightness
  :   5.14

  WS (shortest)
  :   0.95 m/s (-2.04 m/s)
- ID
  :   114273b

  Departure time
  :   2012-06-13 01:05:10 AKDT

  Flight time
  :   26.03 hours

  Flight length
  :   1440.68 km

  Detour
  :   419.38 km

  Track straightness
  :   1.41

  WS (shortest)
  :   0.06 m/s (-5.25 m/s)
- ID
  :   114274

  Departure time
  :   2012-06-02 00:37:00 AKDT

  Flight time
  :   19.58 hours

  Flight length
  :   1049.71 km

  Detour
  :   161.63 km

  Track straightness
  :   1.18

  WS (shortest)
  :   0.32 m/s (-3.39 m/s)
- ID
  :   114275

  Departure time
  :   2012-06-04 00:14:31 AKDT

  Flight time
  :   20.75 hours

  Flight length
  :   1042.48 km

  Detour
  :   95.64 km

  Track straightness
  :   1.1

  WS (shortest)
  :   -2.65 m/s (-3.39 m/s)
- ID
  :   114276

  Departure time
  :   2012-06-03 00:25:01 AKDT

  Flight time
  :   24.25 hours

  Flight length
  :   1601.94 km

  Detour
  :   34.08 km

  Track straightness
  :   1.02

  WS (shortest)
  :   6.93 m/s (6.97 m/s)
- ID
  :   114278

  Departure time
  :   2012-06-03 20:29:27 AKDT

  Flight time
  :   16.49 hours

  Flight length
  :   970.55 km

  Detour
  :   12.42 km

  Track straightness
  :   1.01

  WS (shortest)
  :   -3.35 m/s (-3.29 m/s)
- ID
  :   114279

  Departure time
  :   2012-06-11 19:57:45 AKDT

  Flight time
  :   47.74 hours

  Flight length
  :   2383 km

  Detour
  :   1037.48 km

  Track straightness
  :   1.77

  WS (shortest)
  :   -0.71 m/s (-11.33 m/s)
- ID
  :   114280

  Departure time
  :   2012-06-03 23:07:42 AKDT

  Flight time
  :   29 hours

  Flight length
  :   1386.03 km

  Detour
  :   578.8 km

  Track straightness
  :   1.72

  WS (shortest)
  :   -1.38 m/s (-3 m/s)
- ID
  :   114282

  Departure time
  :   2012-05-31 22:55:33 AKDT

  Flight time
  :   25 hours

  Flight length
  :   1572.39 km

  Detour
  :   27.06 km

  Track straightness
  :   1.02

  WS (shortest)
  :   -0.16 m/s (0.76 m/s)
- ID
  :   114283

  Departure time
  :   2012-06-07 19:11:23 AKDT

  Flight time
  :   32.68 hours

  Flight length
  :   1920.96 km

  Detour
  :   45.88 km

  Track straightness
  :   1.02

  WS (shortest)
  :   3.33 m/s (5.58 m/s)
- ID
  :   114284a

  Departure time
  :   2012-06-04 01:45:05 AKDT

  Flight time
  :   16.75 hours

  Flight length
  :   716.68 km

  Detour
  :   625.06 km

  Track straightness
  :   7.82

  WS (shortest)
  :   -0.14 m/s (0.45 m/s)
- ID
  :   114284b

  Departure time
  :   2012-06-11 22:14:20 AKDT

  Flight time
  :   17.06 hours

  Flight length
  :   1235.35 km

  Detour
  :   298.04 km

  Track straightness
  :   1.32

  WS (shortest)
  :   3.57 m/s (6.51 m/s)
- ID
  :   114285

  Departure time
  :   2012-06-08 20:02:28 AKDT

  Flight time
  :   26.34 hours

  Flight length
  :   1900.06 km

  Detour
  :   52.05 km

  Track straightness
  :   1.03

  WS (shortest)
  :   6.25 m/s (6.76 m/s)
- ID
  :   114286

  Departure time
  :   2012-06-07 22:33:03 AKDT

  Flight time
  :   11.75 hours

  Flight length
  :   812.33 km

  Detour
  :   49.48 km

  Track straightness
  :   1.06

  WS (shortest)
  :   9.84 m/s (11.61 m/s)
- ID
  :   114287

  Departure time
  :   2012-06-03 22:09:56 AKDT

  Flight time
  :   34.01 hours

  Flight length
  :   2009.11 km

  Detour
  :   196.77 km

  Track straightness
  :   1.11

  WS (shortest)
  :   5.51 m/s (8.08 m/s)
- ID
  :   114288

  Departure time
  :   2012-06-07 18:56:15 AKDT

  Flight time
  :   25.25 hours

  Flight length
  :   1831.67 km

  Detour
  :   26.77 km

  Track straightness
  :   1.01

  WS (shortest)
  :   5.5 m/s (6.09 m/s)
- ID
  :   114290

  Departure time
  :   2012-06-03 23:58:50 AKDT

  Flight time
  :   26.83 hours

  Flight length
  :   1600.65 km

  Detour
  :   79.68 km

  Track straightness
  :   1.05

  WS (shortest)
  :   4.73 m/s (5.71 m/s)
- ID
  :   114291

  Departure time
  :   2012-06-04 03:17:48 AKDT

  Flight time
  :   27.6 hours

  Flight length
  :   1537.39 km

  Detour
  :   201.6 km

  Track straightness
  :   1.15

  WS (shortest)
  :   2.63 m/s (3.93 m/s)
- ID
  :   114293

  Departure time
  :   2012-06-08 23:32:59 AKDT

  Flight time
  :   37.25 hours

  Flight length
  :   2132.4 km

  Detour
  :   617.17 km

  Track straightness
  :   1.41

  WS (shortest)
  :   3.33 m/s (9.51 m/s)
- ID
  :   114295

  Departure time
  :   2012-06-08 00:53:52 AKDT

  Flight time
  :   26.75 hours

  Flight length
  :   1548.58 km

  Detour
  :   279.63 km

  Track straightness
  :   1.22

  WS (shortest)
  :   5.04 m/s (9.24 m/s)
- ID
  :   114297

  Departure time
  :   2012-06-10 20:13:13 AKDT

  Flight time
  :   38 hours

  Flight length
  :   2608.79 km

  Detour
  :   2139.33 km

  Track straightness
  :   5.56

  WS (shortest)
  :   4.62 m/s (-16.63 m/s)
- ID
  :   114298

  Departure time
  :   2012-06-07 22:04:10 AKDT

  Flight time
  :   21.54 hours

  Flight length
  :   723.21 km

  Detour
  :   45.07 km

  Track straightness
  :   1.07

  WS (shortest)
  :   -13.83 m/s (-12.26 m/s)
- ID
  :   114299

  Departure time
  :   2012-06-03 22:01:28 AKDT

  Flight time
  :   19.5 hours

  Flight length
  :   1059.61 km

  Detour
  :   110.75 km

  Track straightness
  :   1.12

  WS (shortest)
  :   -2.03 m/s (-3.27 m/s)
- ID
  :   114300

  Departure time
  :   2012-06-07 22:33:27 AKDT

  Flight time
  :   26.9 hours

  Flight length
  :   1710.06 km

  Detour
  :   161.2 km

  Track straightness
  :   1.1

  WS (shortest)
  :   5.64 m/s (7.67 m/s)
- ID
  :   114301

  Departure time
  :   2012-06-08 23:05:51 AKDT

  Flight time
  :   21.08 hours

  Flight length
  :   1433.47 km

  Detour
  :   231.64 km

  Track straightness
  :   1.19

  WS (shortest)
  :   5.5 m/s (11.1 m/s)
- ID
  :   114303

  Departure time
  :   2012-06-11 20:39:05 AKDT

  Flight time
  :   26.75 hours

  Flight length
  :   1192.94 km

  Detour
  :   69.24 km

  Track straightness
  :   1.06

  WS (shortest)
  :   -8.35 m/s (-10.75 m/s)
- ID
  :   114304

  Departure time
  :   2012-06-05 13:19:00 AKDT

  Flight time
  :   55.14 hours

  Flight length
  :   2286.55 km

  Detour
  :   396.07 km

  Track straightness
  :   1.21

  WS (shortest)
  :   4.44 m/s (6.89 m/s)
- ID
  :   114305

  Departure time
  :   2012-06-07 22:32:38 AKDT

  Flight time
  :   26.44 hours

  Flight length
  :   1798.92 km

  Detour
  :   103.23 km

  Track straightness
  :   1.06

  WS (shortest)
  :   4.87 m/s (6.99 m/s)
- ID
  :   114306

  Departure time
  :   2012-06-08 21:11:55 AKDT

  Flight time
  :   21.5 hours

  Flight length
  :   1623.43 km

  Detour
  :   42.35 km

  Track straightness
  :   1.03

  WS (shortest)
  :   7.61 m/s (8.38 m/s)
- ID
  :   114307

  Departure time
  :   2012-06-08 21:56:26 AKDT

  Flight time
  :   37.81 hours

  Flight length
  :   2189.26 km

  Detour
  :   1098.31 km

  Track straightness
  :   2.01

  WS (shortest)
  :   1.33 m/s (-12.48 m/s)
- ID
  :   114308

  Departure time
  :   2012-06-07 20:51:55 AKDT

  Flight time
  :   33.19 hours

  Flight length
  :   1775.87 km

  Detour
  :   37.09 km

  Track straightness
  :   1.02

  WS (shortest)
  :   6.05 m/s (6.76 m/s)
- ID
  :   114309

  Departure time
  :   2012-06-06 08:22:27 AKDT

  Flight time
  :   32.85 hours

  Flight length
  :   2123.77 km

  Detour
  :   159.45 km

  Track straightness
  :   1.08

  WS (shortest)
  :   6.69 m/s (7.62 m/s)
- ID
  :   114310

  Departure time
  :   2012-06-03 23:15:37 AKDT

  Flight time
  :   24.85 hours

  Flight length
  :   1316.09 km

  Detour
  :   108.9 km

  Track straightness
  :   1.09

  WS (shortest)
  :   3.5 m/s (3.65 m/s)
- ID
  :   114311

  Departure time
  :   2012-06-03 22:09:09 AKDT

  Flight time
  :   24.5 hours

  Flight length
  :   1259.07 km

  Detour
  :   1112.73 km

  Track straightness
  :   8.6

  WS (shortest)
  :   -0.03 m/s (0.1 m/s)
- ID
  :   42700

  Departure time
  :   2014-06-09 21:44:25 AKDT

  Flight time
  :   16.04 hours

  Flight length
  :   1122.29 km

  Detour
  :   37.53 km

  Track straightness
  :   1.03

  WS (shortest)
  :   -2.22 m/s (-2.24 m/s)
- ID
  :   43815

  Departure time
  :   2014-06-08 22:06:56 AKDT

  Flight time
  :   19.66 hours

  Flight length
  :   1463.18 km

  Detour
  :   15 km

  Track straightness
  :   1.01

  WS (shortest)
  :   -3 m/s (-3.44 m/s)
- ID
  :   44245

  Departure time
  :   2014-06-10 18:57:48 AKDT

  Flight time
  :   29.71 hours

  Flight length
  :   1303.89 km

  Detour
  :   740.34 km

  Track straightness
  :   2.31

  WS (shortest)
  :   -0.62 m/s (-1.83 m/s)
- ID
  :   46980

  Departure time
  :   2014-06-13 23:07:34 AKDT

  Flight time
  :   19.66 hours

  Flight length
  :   1245.67 km

  Detour
  :   58.15 km

  Track straightness
  :   1.05

  WS (shortest)
  :   1.42 m/s (1.57 m/s)
- ID
  :   48907

  Departure time
  :   2014-06-09 21:38:42 AKDT

  Flight time
  :   20.5 hours

  Flight length
  :   1001.57 km

  Detour
  :   476.91 km

  Track straightness
  :   1.91

  WS (shortest)
  :   0.57 m/s (-3.4 m/s)
- ID
  :   48908

  Departure time
  :   2014-06-08 22:03:01 AKDT

  Flight time
  :   24.75 hours

  Flight length
  :   1595.35 km

  Detour
  :   41.64 km

  Track straightness
  :   1.03

  WS (shortest)
  :   2.39 m/s (1.66 m/s)
- ID
  :   48909

  Departure time
  :   2014-06-08 05:36:27 AKDT

  Flight time
  :   22.03 hours

  Flight length
  :   1227.45 km

  Detour
  :   170.3 km

  Track straightness
  :   1.16

  WS (shortest)
  :   -3.41 m/s (-3.45 m/s)
- ID
  :   48910

  Departure time
  :   2014-06-07 18:29:07 AKDT

  Flight time
  :   21.24 hours

  Flight length
  :   1580.44 km

  Detour
  :   18.77 km

  Track straightness
  :   1.01

  WS (shortest)
  :   3.56 m/s (4.06 m/s)
- ID
  :   48911

  Departure time
  :   2014-06-11 20:23:05 AKDT

  Flight time
  :   33.86 hours

  Flight length
  :   1623.01 km

  Detour
  :   1465.55 km

  Track straightness
  :   10.31

  WS (shortest)
  :   -1.45 m/s (-4.5 m/s)
- ID
  :   49083

  Departure time
  :   2014-06-06 21:12:25 AKDT

  Flight time
  :   24.09 hours

  Flight length
  :   1000.57 km

  Detour
  :   188.01 km

  Track straightness
  :   1.23

  WS (shortest)
  :   -4.51 m/s (-2.41 m/s)
- ID
  :   49793

  Departure time
  :   2014-06-05 22:42:16 AKDT

  Flight time
  :   22.25 hours

  Flight length
  :   1052.55 km

  Detour
  :   8.06 km

  Track straightness
  :   1.01

  WS (shortest)
  :   0.78 m/s (2.11 m/s)
- ID
  :   49796

  Departure time
  :   2014-06-05 07:10:47 AKDT

  Flight time
  :   10.01 hours

  Flight length
  :   684.47 km

  Detour
  :   11.56 km

  Track straightness
  :   1.02

  WS (shortest)
  :   2.87 m/s (2.32 m/s)
- ID
  :   52203

  Departure time
  :   2014-06-06 01:58:05 AKDT

  Flight time
  :   18 hours

  Flight length
  :   977.74 km

  Detour
  :   45.93 km

  Track straightness
  :   1.05

  WS (shortest)
  :   1.59 m/s (2.75 m/s)
- ID
  :   52497

  Departure time
  :   2014-06-08 23:29:24 AKDT

  Flight time
  :   23 hours

  Flight length
  :   1018.61 km

  Detour
  :   91.47 km

  Track straightness
  :   1.1

  WS (shortest)
  :   3.5 m/s (5.57 m/s)
- ID
  :   52552

  Departure time
  :   2014-06-12 00:00:37 AKDT

  Flight time
  :   29.08 hours

  Flight length
  :   1526.71 km

  Detour
  :   750.68 km

  Track straightness
  :   1.97

  WS (shortest)
  :   -1.02 m/s (0.04 m/s)
- ID
  :   53131a

  Departure time
  :   2014-06-10 19:44:07 AKDT

  Flight time
  :   24.55 hours

  Flight length
  :   1140.44 km

  Detour
  :   1038.11 km

  Track straightness
  :   11.14

  WS (shortest)
  :   -0.8 m/s (-1.51 m/s)
- ID
  :   53131b

  Departure time
  :   2014-06-13 21:56:32 AKDT

  Flight time
  :   10.38 hours

  Flight length
  :   700.1 km

  Detour
  :   11.31 km

  Track straightness
  :   1.02

  WS (shortest)
  :   0.85 m/s (-0.06 m/s)
- ID
  :   53247

  Departure time
  :   2014-06-13 04:55:44 AKDT

  Flight time
  :   7.35 hours

  Flight length
  :   583.81 km

  Detour
  :   64.13 km

  Track straightness
  :   1.12

  WS (shortest)
  :   7.32 m/s (5.93 m/s)
- ID
  :   53500

  Departure time
  :   2014-06-07 20:47:15 AKDT

  Flight time
  :   23.47 hours

  Flight length
  :   1612.6 km

  Detour
  :   49.27 km

  Track straightness
  :   1.03

  WS (shortest)
  :   3.67 m/s (3.95 m/s)
- ID
  :   53502

  Departure time
  :   2014-06-05 15:13:40 AKDT

  Flight time
  :   9.25 hours

  Flight length
  :   642.28 km

  Detour
  :   13.58 km

  Track straightness
  :   1.02

  WS (shortest)
  :   5.05 m/s (4.65 m/s)
- ID
  :   53504

  Departure time
  :   2014-06-08 23:40:54 AKDT

  Flight time
  :   20.13 hours

  Flight length
  :   1161.16 km

  Detour
  :   64.47 km

  Track straightness
  :   1.06

  WS (shortest)
  :   -4.23 m/s (-4.35 m/s)
- ID
  :   53815

  Departure time
  :   2014-06-05 14:53:17 AKDT

  Flight time
  :   22.7 hours

  Flight length
  :   1060.83 km

  Detour
  :   62.69 km

  Track straightness
  :   1.06

  WS (shortest)
  :   1.19 m/s (1.89 m/s)
- ID
  :   53817

  Departure time
  :   2014-06-06 05:10:51 AKDT

  Flight time
  :   24.34 hours

  Flight length
  :   1289.35 km

  Detour
  :   91.3 km

  Track straightness
  :   1.08

  WS (shortest)
  :   0.47 m/s (0.92 m/s)
- ID
  :   53820a

  Departure time
  :   2014-06-17 23:35:51 AKDT

  Flight time
  :   20.17 hours

  Flight length
  :   972.35 km

  Detour
  :   828.43 km

  Track straightness
  :   6.76

  WS (shortest)
  :   2.68 m/s (0.89 m/s)
- ID
  :   53820b

  Departure time
  :   2014-06-23 23:41:34 AKDT

  Flight time
  :   15.4 hours

  Flight length
  :   1208.38 km

  Detour
  :   99.16 km

  Track straightness
  :   1.09

  WS (shortest)
  :   6.88 m/s (10.39 m/s)
- ID
  :   53821

  Departure time
  :   2014-06-06 05:11:42 AKDT

  Flight time
  :   15.25 hours

  Flight length
  :   701.26 km

  Detour
  :   118.36 km

  Track straightness
  :   1.2

  WS (shortest)
  :   0.59 m/s (0.49 m/s)
- ID
  :   53823

  Departure time
  :   2014-06-06 22:52:54 AKDT

  Flight time
  :   25.67 hours

  Flight length
  :   963.39 km

  Detour
  :   161.9 km

  Track straightness
  :   1.2

  WS (shortest)
  :   -1.5 m/s (-1.94 m/s)
- ID
  :   53830

  Departure time
  :   2014-06-08 00:42:35 AKDT

  Flight time
  :   14.2 hours

  Flight length
  :   984.27 km

  Detour
  :   20.19 km

  Track straightness
  :   1.02

  WS (shortest)
  :   -2.75 m/s (-4.2 m/s)
- ID
  :   54042

  Departure time
  :   2014-06-08 23:42:48 AKDT

  Flight time
  :   20.01 hours

  Flight length
  :   893.09 km

  Detour
  :   145.76 km

  Track straightness
  :   1.2

  WS (shortest)
  :   2.02 m/s (4.45 m/s)
- ID
  :   54054

  Departure time
  :   2014-06-05 14:59:33 AKDT

  Flight time
  :   20.67 hours

  Flight length
  :   1021.71 km

  Detour
  :   57.44 km

  Track straightness
  :   1.06

  WS (shortest)
  :   2.08 m/s (3.46 m/s)
- ID
  :   54568

  Departure time
  :   2014-06-05 20:35:39 AKDT

  Flight time
  :   28.5 hours

  Flight length
  :   1494.1 km

  Detour
  :   16.51 km

  Track straightness
  :   1.01

  WS (shortest)
  :   0.26 m/s (0.69 m/s)
- ID
  :   56512

  Departure time
  :   2014-06-08 20:26:32 AKDT

  Flight time
  :   28.81 hours

  Flight length
  :   1707.66 km

  Detour
  :   126.5 km

  Track straightness
  :   1.08

  WS (shortest)
  :   1.79 m/s (1.9 m/s)
- ID
  :   59122

  Departure time
  :   2014-06-08 21:20:01 AKDT

  Flight time
  :   16.69 hours

  Flight length
  :   1119.09 km

  Detour
  :   31.91 km

  Track straightness
  :   1.03

  WS (shortest)
  :   -3.33 m/s (-4.43 m/s)
- ID
  :   59254

  Departure time
  :   2014-06-09 01:19:54 AKDT

  Flight time
  :   16.75 hours

  Flight length
  :   1080.79 km

  Detour
  :   4.97 km

  Track straightness
  :   1

  WS (shortest)
  :   -4.37 m/s (-4.37 m/s)
- ID
  :   59257

  Departure time
  :   2014-06-09 00:01:18 AKDT

  Flight time
  :   19.29 hours

  Flight length
  :   884.96 km

  Detour
  :   126 km

  Track straightness
  :   1.17

  WS (shortest)
  :   2.53 m/s (4.58 m/s)
- ID
  :   59987

  Departure time
  :   2014-06-09 21:03:51 AKDT

  Flight time
  :   24 hours

  Flight length
  :   1360.02 km

  Detour
  :   94.44 km

  Track straightness
  :   1.07

  WS (shortest)
  :   2.05 m/s (3.07 m/s)
